# Thyrotomy

**Published:** 1877-01

**Authors:** H. A. Johnson

**Affiliations:** Prof. of Principles and Practice of Medicine, Chicago Medical College; Chicago


### Chicago Medical Journal

AND

### EXAMINER.

Vol. XXXIV.—JANUARY, 1877.—No. 1.

### Original Communications.

### THYROTOMY: WITH REPORTS OF FOUR OPERATIONS.

By H. A. JOHNSON, M.D.

Prof. of Principles and Practice of Medicine, Chicago Medical College.

(Read before the Chicago Medical Society, December 4, 1876.)

The operation of thyrotomy was, so far as I am able to learn, first performed by Brauers, of Louvain, in 1833. I am unable to say with certainty how many times it has been performed since, but I find records of only fifty-nine cases. It is to be presumed that some have been published of which I have no knowledge. Dr. Morel Mackenzie, in 1873, knew of only forty-eight cases, which he tabulates with reference to date, operation, age, sex, symptoms, mortality, respiration, voice, recurrence or incomplete removal. As to result, he estimates: Complete success in 14.58 per cent.; partial success in 22.91 per cent.; deaths, 8.33 per cent.; severe dyspnœa, requiring cannula, 31.25 per cent.; severe dyspnœa, requiring fresh operation, 8.33 per cent. He found aphonia in 40 per cent.; dysphonia in 20 per cent.; modified voice in 11.11 per cent.; not stated, but probably defective voice, 6.66 per cent.; recurrence or incomplete removal, 38.46 per cent. His conclusions are as follows:

"1. That the operation ought never to be performed for loss of voice alone.

"2. That in cases of cancer the operation is useless except where the growth is very small and distinctly circumscribed.

"3. That the operation should be confined to those cases in which there is danger to life from suffocation or dysphagia, and then only to be performed after an experienced laryngoscopist has pronounced it impossible to remove the growth *per vias naturales.*" See his reply to Mr. Durham, London, 1873.

Mr. Durham had taken a much more hopeful view of the operation. He says:

"*First*—That the dangers and difficulties attending it are neither so numerous nor so considerable as have been represented and commonly supposed.

"*Second*—That the success hitherto achieved has been so marked and so indisputable as to justify and encourage in any such case as may seem appropriate, an earlier, bolder and more ready resort to this method than has hitherto prevailed."—55*th vol. Med. Chirurg. Tras., London.*

Of Mackenzie's forty-eight cases, nine were performed in America, namely: By Dr. Gurdon Buck, four cases; by Dr. Sands, one case; by Dr. Gouley, two cases; by Dr. Cohen, one case; by Dr. Cutter, one case.

Navratil, in a Vienna medical journal, published in 1875, reports twelve cases with good results. Three cases by this operator are included in Mackenzie's tables, and of these three one is reported aphonic and the other two dysphonic. In the paper referred to twelve cases are reported, and in all of them the voice was restored. I have not seen the original report, but a notice of it in the *Revue des Sciences Médicales*, Vol. IV., p. 638, Paris. Do the twelve cases reported in 1875 include the three in Mackenzie's paper? I assume that they do. In Mackenzie's table there is a case by Krishaber, in 1869, in which the voice is reported normal.

In the *Dictionnaire des SciencesMédicales*, under the article Larynx, Krishaber and Peter state that the voice is always extinct after the separation of the thyroid cartilages. In Krishaber's case, in Mackenzie's table, the voice is reported normal. The article in the *Dictionnaire* was published after the report of the case. I presume, therefore, we may infer that the voice did not remain normal. Of Navratil's twelve cases, in all of which he reports the voice restored, he says that two were able to sing. In the other cases, while the voice was restored, there was probably some defect either in flexibility or quality.

Dr. George M. Lofferts, of New York, reports a case of thyrotomy for the removal of both ventricles, which were prolapsed. The voice was not destroyed by the operation.—*Ann. des Mal. de l'Oreille et du Larynx.*

J. Bœckel performed the operation for removal of a tumor from the right ventricle, probably prolapse. The voice was not destroyed.—*Gazette Méd. de Strasbourg.*

Of the fifteen cases of which I have some knowledge since Mackenzie's table was published in 1873, only one has died. In thirteen the voice has been restored, or at least not destroyed.

Bœckel suggests that in operating for thyrotomy the head should be low, so that the blood will gravitate toward the mouth rather than toward the trachea. This suggestion seems to me to be worthy of consideration.

Among those who have written upon the subject there seems to be a wide difference of opinion as to the necessity, the methods, and the results of the operation. Some authorities insist that all, or nearly all, growths can and ought to be removed *per vias naturales;* others, with Mr. Durham, that thyrotomy should be resorted to more boldly and more readily.

It is insisted by some that the cricoid should never be divided; others assert that the separation of this cartilage gives rise to no inconvenience. Dr. Mackenzie and Mr. Durham do not by any means arrive at the same conclusions as to the results. Mr. Durham finds complete success in 51.35 per cent. of the cases analyzed; Dr. Mackenzie, from a study of the same histories, finds complete success in only 14.58 per cent.

Mr. Lennox Browne, in a communication read before the Medical Society of London, upon the treatment of non-malignant growths in the larynx, presented the following general propositions, which are of interest in connection with this subject:

"1. Attempts made to remove excrescences of the larynx by ablation are not as harmless as is generally supposed.

"2. The symptoms occasioned by benign tumors of the larynx are not marked enough to necessitate the use of instruments.

"3. Many of these growths are destroyed, or are reduced, by local treatment or appropriate general treatment, especially where they are recent.

"4. The reappearance of laryngeal tumors after their ablation by *per vias naturales*, is more frequent than one would suppose.

"5. The transformation of benign into malignant growths is a quite frequent result of attempts at extraction.

"6. The instruments more frequently used at the present day are much more dangerous than those formerly employed. They determine perichondritis by wounding the sound parts.

"7. As to extra laryngeal operations, they should be reserved for those cases where there is danger of asphyxia or dysphagia." —*British Med. Journal*, *May*, 1875; *from Annales des Maladies de l'Oreille et du Larynx*, *Aug.*, 1876.

The recorded cases in which the details and results are fully given are probably not yet sufficiently numerous to justify a positive judgment upon many of the questions involved.

As some contribution to the history and literature of this subject, I beg to submit to the Society reports of four operations of thyrotomy — the only ones, so far as I know, performed in Illinois. Two of these operations were upon the same patient; but the circumstances were such as to justify separate histories. These four, with the nine of Navratil, one by Lofferts, and one by Boeckel, added to the forty-eight tabulated by Mackenzie, make in all sixty-three cases:

Case I. George Mundie, aged ten years, of English parentage, was referred to me by Dr. E. P. Cook, of Mendota, Ill. I saw him first December 4, 1868. He was well grown for his age, and appeared healthy. His mother stated that he had been somewhat hoarse from infancy; had not been subject to croup, but for the last three years had been, with the exception of two short intervals, aphonic. Before the voice became extinct, three years ago, he took a bad cold, and coughed severely for five or six weeks. The loss of voice was gradual. About two years ago he again took a severe cold, and after three or four days the voice returned, and was quite natural for ten days, when it began to fail, and became entirely extinct as he recovered from his cold. About eight months ago the voice returned for a few days, while suffering from a severe cold, but was not natural in quality. Upon examination I found an uneven strawberry-like growth, of a bright rose color, between the anterior portion of the cords, and apparently attached beneath them. The posterior extremities of the cords were free. There was more difficulty in expiration than in inspiration. The case was kept under observation for some time, and repeated efforts made to remove the growth with the forceps, but without success. Repeated subsequent examinations confirmed the diagnosis of a tumor of considerable size located in the infra-glottic space. It could be thrown up between the cords by coughing, and would then be held there by the cords. There was constant difficulty of breathing, and attacks of great dyspnœa became quite frequent. Finding it impossible to remove it by the natural passages, as it would fall down and out of the reach of instruments upon each effort, while spasm of the larynx was easily provoked, I advised thyrotomy. The operation was agreed to, and a day set for its performance; but the little fellow in the meantime fell into the hands of a quack, who scouted the idea of a tumor and promised to cure him in three weeks. I did not see him again; but I advised my friend, Dr. Cook, who had in the meantime acquired a facility in the use of the laryngoscope, and who had taken much interest in the case, to be prepared to perform tracheotomy if necessary, as I feared it soon would be, and suggested that in that event, if the parents would consent to it, he open the larynx and remove the tumor.

In December, 1870, I received from the doctor a letter, of which the following is a copy:

"Mendota, Ill., December 16, 1870.

"H. A. Johnson, M.D.: *Dear Sir*—I have no doubt you remember, and that it will give you pleasure to hear from your former patient, Master G—— M——. I performed the operation of thyrotomy in his case seven days since. He is doing finely. There proved to be a double mass of nearly equal size, with a common pedicle, or I might say neither strictly pedunculated nor sessile; the upper mass nearly spheroid in shape half inch in diameter; the lower, and which was with difficulty seen by the laryngoscope, oblong and flattened—greatest diameter five-eighths of an inch. Nothing could more perfectly resemble a diminutive cauliflower. Of its benignancy I hope there can be no question. The operation was not very difficult, but required more than two hours' time. External incision two and a half inches long, from a little above the hyoid bone to below the cricoid cartilage, but kept clear of the isthmus of the thyroid body, and did not expose the rings of the trachea, as a precautionary step, in view of the possible necessity for the introduction of the tube. Made the external incision a little to one side of the median line, and dissected down obliquely to the larynx, etc. Thus, upon closing the external wound, had more tissues upon the one side, and by that means steadied the thyroid cartilage and perfectly controlled the over-riding of the one section upon the other. But I need not go into particulars, as they are no doubt more familiar to you than to me. I expect in a few days to have a laryngoscopic view of the parts.

"I am, yours truly, E. P. Cook."

I have heard from this case repeatedly since; and in June, 1874—more than three and a half years after the operation—Dr. Cook told me that there had been no return of laryngeal trouble, and that the voice was quite natural. This, so far as

I know, is the first case of thyrotomy performed west of Philadelphia and New York.

The following case was for some weeks under my care, and I made repeated efforts to destroy the growth by the galvano-cautery. After one of the applications he became frightened, and I did not see him again until the operation for thyrotomy. From Dr. E. Bert, of this city, who performed the operation, I have the following statement:

"CHICAGO, November 27, 1876.

"DR. H. A. JOHNSON, CITY: *Dear Doctor*—I copy from my memorandum of the case: Carl Moll, forty-five years of age (1874), merchant tailor. Never sick before; first symptoms of tumor-laryngis May, 1873; slight irritation in swallowing, particularly of dry substances; beginning hoarseness at same time. During the summer of 1873 his symptoms gradually increased in intensity, until in autumn complete aphonia set in. The pains appear at the same place—on the left side of os hyoid and underneath—radiating often, and mainly at nights, toward left ear. Pain increased while swallowing, sometimes so intense that patient refuses to eat.

"*Coughed* very little in the beginning of the disease; now of a dry and hacking character, accompanied quite recently by pain. Expectoration, of a slimy and salivary character, quite copious.

"*Alteration* of voice has been almost his first symptom. Up to the middle of last winter it was sometimes better than at others; since about six months it has failed almost completely.

"*Dyspnœa* begins to appear now, and alarms patient more than anything else. The copious collection of mucus forces him to spit quite often, and at such times he cannot breathe quite freely. In the month of June (1874) there was quite an inflammatory swelling, caused by the continued use of the galvano-cautery, and then he was suffering from dyspnœa more than even now (November, 1874). He is able to relieve himself from the trouble of dyspnœa, by rolling over to his right side, which seems to indicate the mobility of the tumor.

"*Dysphagia.* Patient is able to swallow all kinds and form of nourishment. The act of swallowing is painful, and followed by an attack of cough generally, by which small masses of edibles are thrown back. This symptom is varying at different times.

"*Pain* has existed from the beginning, and was of a constant, dull character. He thought "he could cough out the foreign substance." Pain has gradually increased in intensity, it being unusually sharp now. It is confined to the left side of the os hyoid, and extends upward to the left ear, which is sometimes the principal seat of complaint, particularly at nights.

"June, July and August all possible alteratives were applied, and finally galvanic treatment was instituted.

"*November* 19, 1874. Great emaciation during eight days; loss of strength; eats almost nothing. The tumor very much swollen; a small ulceration to be seen on left half of epiglottis. Sputa very copious, partly of a bad odor. *Wants* operation at all hazards. Low tracheotomy performed November 20, 1874.

"Thyrotomy was performed November 22, 1874, the cartilage being very resistant; there was considerable difficulty in splitting it open. Another trouble was the lack of a convenient instrument to keep both halves of the cartilage and larynx apart. The tumor was seized with a strong vusella and twisted a number of times around its small base, from which it protruded downward over one and a half inches in the shape of a pear. There was little hæmorrhage. The base of the tumor was strongly touched with perchloride of iron. The patient had a kind of fainting spell soon after the operation, but rallied from it soon with the aid of beef tea and stimulants. He was able to read and write about an hour and a half afterward, and whispered without any pain; could be easily understood. Every possible precaution as regards temperature of room and ventilation and nourishment was very strictly taken; the most accurate scrutiny would fail in finding any omission. The wound seemed to do well. Cleaning the cannula could be done without any trouble; scarcely any fever, no symptoms of pyæmia, gangrene, pneumonia, etc. He continued to do well, and the most sanguine expectations about his final and speedy recovery seemed justified. November 25, 10 P. M., he was in good spirits, not complaining of anything, except he sat up and wrote on a slip of paper: My head feels very sore. At 2 A. M. he was seen, and talked a few words quite intelligently. At 4 A. M., November 26, 1875, he expired, a few moments after he had been watched and found well, without any struggle.

"A *post-mortem* examination made of the larynx failed to discover any local *causa mortis.* The wound was in a good condition, no gangrenous appearance or (diphtheric?) exudation, no signs of hæmorrhage."

Case III. Viola Franks, female, aged six and a half years, was brought to me by her mother, in the fall of 1873. She had whooping cough in the winter of 1872–3, and as she recovered from it her voice began to be hoarse and finally became extinct. At the time of consultation she was apparently in good health, with the exception of a slight cough and the aphonia, with occasional paroxysms of dyspnœa. Her appetite was fair, and she was well nourished. She was bright and cheerful, her cheeks ruddy and her lips red. Upon examination I found the epiglottis narrow and rolled like a dried leaf; it was dependent, and covered over the entrance to the larynx so that I could not see the vocal cords. The color of the parts, so far as I could observe them, was normal.

During the winter of 1873–4 I saw her several times, but could never see the glottis. Her general health failed a little, and in the spring became decidedly impaired. She was placed upon tonics, which for a while seemed to benefit her; but in the early summer of 1874 dyspnœa, both upon inspiration and expiration, became constant, with frequent spasms of the glottis. She was emaciated, pale, appetite poor, and all the symptoms so alarming, that on the fifth of July I performed high tracheotomy. She immediately began to improve, and gained in flesh and strength. Early in September she again began to fail; the appetite was poor, and there was difficulty in swallowing, apparently from pressure upon the œsophagus. So far I had not been able to see below the glottis, and only occasionally to get a glimpse of the vocal cords. The epiglottis rested back, covering in the laryngeal cavity, and it was only by an effort at coughing that it would be thrown up. She would not tolerate in the pharynx the presence of a hook, so that I could not raise it. From the fact that there was no sufficient cause for the dyspnœa above the cords, and especially no impediment above them to expiration, and from the fact that there was no mechanical obstruction below the cricoid, I was confirmed in the opinion which I had previously held, that there was a tumor in the infra-glottic space of such size as to mechanically obstruct the passage of air. The dysphagia seemed to be caused by pressure of the tumor upon the parts posterior to the larynx.

September 27, 1874, with the assistance of Drs. Norcom and Sherman, I performed thyrotomy, dividing the soft parts in the median line, opening the larynx through the crico-thyroid membrane, introducing a grooved director upwards and between the vocal cords, and then dividing the cartilage. Upon separating the parts I found a tumor completely filling the space below the glottic chink and attached to the walls upon the right side of the space. The attachment was large, and its thorough removal somewhat difficult, as it extended below the superior border of the cricoid and up to the right cord. In order to make the removal as thorough as possible, I divided the cricoid cartilage. The growth was removed partly with the forceps and partly with a pair of sharply curved scissors. There was considerable hæmorrhage, which was controlled by the application of a solution of persulphate of iron. The base was cauterized with nitrate of silver, and the parts brought together. There seemed to be no tendency to displacement, and I simply closed the soft parts with two interrupted sutures, and supported them with strips of adhesive plaster. The operation was well borne, and was followed by very little fever or discomfort. In a few days she was up and around the house, and soon recovered her general health. The cannula was however allowed to remain.

The tumor was irregular in shape, of a cauliflower appearance, and about five-eighths of an inch in its transverse diameter, and extending from the vocal cords down to the lower border of the cricoid cartilage. It was soft, and easily broke down under the instruments. A microscopic examination was made, and it was found to consist of epithelial cells with in its interior a basis of connective tissue and blood-vessels. The larger portion of it, however, was made up of rounded or flattened epithelial cells and those not confined to its exterior. It evidently belonged, I think, to the epitheliomas, although upon its surface it presented the ordinary appearance of papilloma in the larynx. She breathed easily through the larynx, and for some weeks I hoped the operation would be followed by complete recovery. She was able to speak aloud, but with difficulty, as I was told; I did not hear her.

In about six weeks her mother thought she did not breathe quite as easily through the larynx; and upon examination, December 1, 1874, I found that she could not breathe at all through the larynx. The tumor was evidently reproduced. Her general health was, however, fair, and continued good during the winter of 1874–5. Portions of the growth came down so that they could be reached through the opening in the trachea, and were removed for examination. The microscopic characters were the same as those of the tumor.

In Dr. Mackenzie's paper, in reply to Mr. Durham, he says: "In all instances where thyrotomy has been performed afresh, the original wound having completely healed up, the operation has been considered as a new case." In my little patient the original wound had completely healed in a very short time after the operation. The respiration through the larynx was good, and the voice had been partially restored. The case was unsuccessful, however, as recurrence took place. In accordance with Dr. Mackenzie's rule, it should now be treated as a new case.

Case IV. The patient continued in fair health during the spring of 1875, but in the early summer began to lose flesh, became pale and anæmic. There began to be some dysphagia, and I determined to repeat the operation. On the 29th of July, 1875, assisted by Drs. Norcom, Freer and Andrews, I again performed thyrotomy, dividing both cartilages from below upwards, having passed a director between the vocal cords before completing the section of the thyroid. The parts were separated, and held apart by two double hooks.

The tumor, upon opening the larynx, immediately protruded through the wound, presenting the appearance of a cauliflower of a pinkish color. The attachment was the same as in the former operation, but somewhat more extensive, reaching further to the left of the median line. The growth was larger than that removed in the previous operation, and was evidently producing a good deal of pressure upon the walls of the larynx. It was carefully and thoroughly detached, and the base touched with strong nitric acid. The section was found to have been made exactly in the median line, between the anterior attachments of the cords, and they were both uninjured and normal throughout their entire extent.

The operation was followed by almost no fever, or any untoward symptom. Even the next day she sat up, and had a fair appetite; swallowed well; and in a week she was out of the house, and the wound had almost entirely healed. As in the former case. she continued to wear the tube in the trachea. From this time on her general health was good, and she breathed easily through the larynx. The inner as well as the outer tube was fenestrated, and she wore a cork much of the time in the cannula.

In March, 1876, eight months after the operation, she began to speak aloud, and for the first time I heard her voice. I removed the tube, as I found no evidence of a recurrence. From that time to the present — December 4, 1876 — her general health has been good. She has been in school during the summer and fall, and habitually speaks in a clear, ringing tone. I find that the voice has a good degree of flexibility, and she has been, during the fall, taking lessons in singing.

The opening in which the cannula was worn is still not quite closed; and for the last few weeks I have touched the parts with solid nitrate of silver. The skin seems to be inverted, keeping the free edges a little apart. There is no purulent discharge from it, only at times a little mucus appearing at the point. It seems now to be rapidly closing.

During the last few months I have repeatedly seen the vocal cords in the laryngeal mirror. They are upon the same plane, normal in color, and smooth and entire throughout their whole extent, with the exception of a very slight roughness near the anterior attachment of the left. The epiglottis is more erect, and the examination is not difficult. It is perhaps too soon to pronounce positively as to a recurrence; but the case seems to be a complete success. The patient was present and was examined by the members of the society. A portion of the tumor with a microscopic section was also exhibited.

CHICAGO, December 4, 1876.

